# Prognostic Factors in High Grade Osteosarcoma Patients Who Received Neoadjuvant Therapy and Subsequently Underwent Surgery: Data from the Turkish Oncology Group

**DOI:** 10.3390/jcm14062024

**Published:** 2025-03-17

**Authors:** Nadiye Sever, Fatih Şimşek, İlknur Deliktaş Onur, Hayati Arvas, Tural Guliyev, Teoman Şakalar, Ceren Mordağ Çiçek, Seval Orman, Emine Bihter Çetin, Kamil Kayaş, Sinem Akbaş, Yeşim Ağyol, Ali Kaan Güren, Pınar Erel, Erkam Kocaaslan, Burak Paçacı, Mustafa Alperen Tunç, Abdussamet Çelebi, Nargiz Majidova, Ayşe Durnalı, Melih Şimşek, Mustafa Şahbazlar, Selver Işık, Rukiye Arıkan, Özlem Ercelep, Murat Sarı, Osman Köstek, İbrahim Vedat Bayoğu

**Affiliations:** 1 Division of Medical Oncology, Department of Internal Medicine, Marmara University Faculty of Medicine, İstanbul 34854, Turkey; yesimagyol@gmail.com (Y.A.); alikaanguren@gmail.com (A.K.G.); pnarerell@gmail.com (P.E.); erkamkocaaslan@gmail.com (E.K.); drpacaci@gmail.com (B.P.); m.alperen.tunc@gmail.com (M.A.T.); abdussametcelebi@gmail.com (A.Ç.); nergiz.mecidova1991@gmail.com (N.M.); dr-selver83@hotmail.com (S.I.); dr_rukiyearikan@hotmail.com (R.A.); ozlembalvan@yahoo.com (Ö.E.); dr.vebay@gmail.com (İ.V.B.); 2 Department of Internal Medicine, Marmara University Faculty of Medicine, İstanbul 34854, Turkey; drfatihsimsek.bjk@gmail.com; 3 Dr. Abdurrahman Yurtaslan Ankara Oncology Training and Research Hospital, Ankara 06200, Turkey; ilknurdeliktas382@gmail.com (İ.D.O.); aysadurnali@gmail.com (A.D.); 4 Dicle University Faculty of Medicine, Diyarbakır 21300, Turkey; hayatiarvas65@gmail.com; 5 Division of Medical Oncology, Bezmialem Vakıf University Hospital, İstanbul 34093, Turkey; dr.t.quliyev@gmail.com (T.G.); mdmelih@gmail.com (M.Ş.); 6 Division of Medical Oncology, Necip Fazıl City Hospital, Kahramanmaraş 46050, Turkey; drteomansakalar@gmail.com; 7 Division of Medical Oncology, Pamukkale University Hospital, Pamukkale 20160, Turkey; cerenmordag@gmail.com; 8 Division of Medical Oncology, Kartal Training and Research Hospital, İstanbul 34865, Turkey; drseval1988@gmail.com; 9 Division of Medical Oncology, Celal Bayar University Hospital, Manisa 45030, Turkey; drbihtereniseler@gmail.com (E.B.Ç.); m_sahbazlar@hotmail.com (M.Ş.); 10 Gaziantep University Faculty of Medicine, Gaziantep 27410, Turkey; kamkay_27@hotmail.com; 11 Koç University Faculty of Medicine, İstanbul 34450, Turkey; sinem_kocak@yahoo.com; 12 Medical Oncology, Department of Internal Medicine, İstanbul Medipol University, İstanbul 34810, Turkey; drmuratsari@gmail.com (M.S.); osmankostek@yahoo.com (O.K.)

**Keywords:** osteosarcoma, neoadjuvant chemotherapy, survival analysis, prognostic factors, tumor necrosis

## Abstract

**Background:** Osteosarcoma is a rare but aggressive bone malignancy. Despite advances in multimodal therapy, survival remains suboptimal, highlighting the need for prognostic markers to guide treatment. **Methods:** This study included 162 osteosarcoma patients who received neoadjuvant chemotherapy followed by surgery between January 2009 and March 2024. Patients received either double (cisplatin + doxorubicin) or triple (MAP or PEI) chemotherapy. Survival analyses were conducted using Kaplan–Meier curves, log-rank tests, and Cox proportional hazards models. **Results:** The median age was 20 years (IQR: 18–29), and 53.1% were male. Patients who received triple chemotherapy regimens demonstrated significantly longer overall survival (OS) compared to those on doublet regimens. High tumor necrosis rates (>90%) and negative surgical margins were strongly associated with improved OS, while metastatic disease at diagnosis, elevated alkaline phosphatase (ALP), and male gender were linked to poorer survival. Multivariate analysis identified adjuvant therapy, age under 18, high necrosis rate, negative margins, and normal ALP as significant OS predictors. **Conclusions:** Triple-agent chemotherapy, necrosis rate ≥90 and negative surgical margins are strongly associated with prolonged survival in osteosarcoma. The key prognostic indicators such as ALP levels, surgical margins and age at diagnosis should guide personalized treatment strategies to improve outcomes in curable patients.

## 1. Introduction

Primary bone cancers are extremely rare neoplasms accounting for ~0.2% of all cancers, and their true incidence is difficult to determine due to the rarity of these tumors [1]. Osteosarcoma is the most common primary malignant bone tumor in children and young adults [2]. The incidence of osteosarcoma has a bimodal age distribution. Under 20 years of age and over 65 years of age is the most common bone tumor [3]. Approximately 10–20% of osteosarcoma patients have metastases at diagnosis, most commonly to the lungs (more than 70%) and rarely to other bones or organs [4]. Conventional osteosarcomas constitute the majority of cases [5]. Conventional osteosarcomas are divided into osteoblastic (76–80%), chondroblastic (10–13%), and fibroblastic (10%) subtypes [6]. Despite histological differences, they have similar clinical behavior and treatment.

Treatment of osteosarcoma is primarily based on neoadjuvant and adjuvant chemotherapy and surgical resection [7]. Distant metastases developed during the course of disease are the main prognostic factors affecting non-metastatic patients’ survival rate, highlighting the substantial role of chemotherapy in eliminating micro metastases. While a methotrexate + doxorubicin and cisplatin (MAP) regimen is recommended for children and adolescents, a doxorubicin + cisplatin regimen is mostly preferred in adults [8,9]. The triple therapy regimen of cisplatin plus ifosfamide and etoposide (PEI) is also preferable in both age groups. Good histopathologic response (≥90% necrosis) to neoadjuvant chemotherapy (NAC) has been shown to be predictive of survival regardless of the type of chemotherapy administered after surgery [10]. Building on the existing literature, this study aimed to assess the effectiveness of different neoadjuvant chemotherapy regimens in patients with osteosarcoma who underwent surgical resection. In addition to evaluating the impact of chemotherapy regimens on survival, we also investigated the prognostic significance of age, gender, and histopathologic characteristics, including tumor necrosis rate and histologic subtype.

## 2. Material and Methods

**Figure A1:**
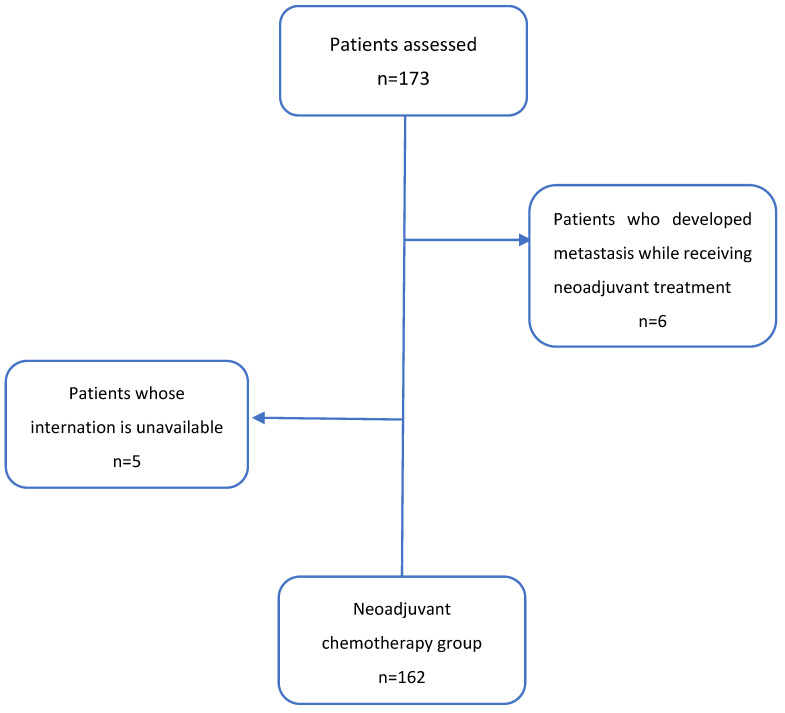
Inclusion criteria of study patients.

Patients with osteosarcoma who were diagnosed between January 2009 and March 2024, received neoadjuvant chemotherapy, and subsequently underwent surgery were included in the study. All osteosarcoma subtypes classified as high-grade in pathology reports were included, without excluding specific subtypes such as high-grade surface osteosarcoma, small cell osteosarcoma, telangiectatic osteosarcoma, or periosteal osteosarcoma. There were 6 patients who developed metastasis and could not be operated on while receiving neoadjuvant therapy and 5 patients whose information could not be reached (total 11 patients) were excluded from the study. A total of 162 patients from 10 centers were analyzed (Appendix A Figure A1). Patients under 18 years of age who were diagnosed and treated in pediatric oncology and whose files were transferred to the adult oncology outpatient clinic during follow-up were also included. The study was approved by the Clinical Research Ethics Committee of Marmara University Faculty of Medicine with the protocol code 22.04.2024.495 on 25 May 2024. It was conducted in accordance with the ethical standards of the Declaration of Helsinki. Due to the retrospective nature of the study, the need for informed consent was waived. Age, gender, Eastern Cooperative Oncology Group Performance Status (ECOG-PS), primary tumor site, tumor size, surgical margin, chemotherapy regimen used in neoadjuvant and adjuvant treatment, presence of metastasis at presentation or follow-up, whether metastasectomy was performed, pathological response and necrosis rate, type of progression (local recurrence and/or metastasis), and death dates were screened. Recurrence was defined as the re-emergence of tumor at the primary site following definitive treatment, whereas distant metastasis referred to the spread of malignant cells to distant organs, most commonly the lungs. Patients received a double or triple chemotherapy regimen as neoadjuvant treatment. The selection between double and triple chemotherapy regimens was influenced by the patient’s age and performance status, as older patients or those with lower functional capacity were more likely to receive double chemotherapy. Additionally, the experience of the clinician at the treatment center played a role in treatment decisions, reflecting institutional preferences and varying levels of expertise in managing osteosarcoma cases. Adjuvant chemotherapy regimens were generally consistent with the neoadjuvant regimens. However, in 9 patients who had received cisplatin + doxorubicin as neoadjuvant chemotherapy and had a tumor necrosis rate of <90%, ifosfamide was added to the adjuvant regimen based on institutional treatment protocols and physician discretion. A pathological complete response was defined as the absence of any residual tumor tissue in the surgical specimen obtained following neoadjuvant chemotherapy or the presence of 1% residue. Elevated alkaline phosphatase (ALP) and lactate dehydrogenase (LDH) were defined as values above the reference range in our hospital. The relationship between the data obtained and disease-free survival (DFS) and overall survival (OS) was analyzed. DFS was calculated from the date of surgery until the date of disease recurrence or last follow-up, as applicable. OS included the date of diagnosis until the date of death from any cause, or the last follow-up for surviving patients.

### Statistical Analysis

Data analysis was performed using SPSS 26.0 statistical software. Continuous data were summarized as median and interquartile range, while categorical variables were analyzed using the Chi-square or Fisher’s exact test. Survival curves were generated using the Kaplan–Meier method for each subgroup, with 95% confidence intervals (CIs). The log-rank test was used to compare differences in survival between groups. Prognostic factors were examined using univariate analysis, with subsequent examination of factors with a *p*-value of less than 0.5 in the multivariate analysis. Hazard ratios (HRs) for these comparisons were calculated using a Cox proportional hazards model. Statistical significance was established at *p* < 0.05.

## 3. Results

### 3.1. The Study Population’s Demographic and Clinical Characteristics

The median age of patients was 20 years, with an interquartile range of 18 to 29 years. The cohort included 76 females (46.9%) and 86 males (53.1%). The majority of the patients (98.8%) had an ECOG-PS score of 0–1. Twenty-six (16%) of the patients were under 18 years of age at diagnosis. The majority of patients (92.6%) had tumor grade 3 at diagnosis. The most common histologic subtypes were osteoblastic (40.7%) and chondroblastic (28.4%). The most common tumor site was the lower extremity (83.3%), and the most common locations were femur (48.1%) and tibia (22.1%). Fourteen (8.6%) patients had pelvic localization. There were 49 (30.2%) patients whose tumor size was over 8 cm. The most commonly used neoadjuvant treatment options were cisplatin + doxorubicin doublet chemotherapy regimen (58%), epirubicin + cisplatin + iphosphamide (19.8%), and doxorubicin + cisplatin + methotrexate (16.7%) triplet chemotherapy regimens. Among the 162 patients who underwent surgery, 147 (90.7%) underwent limb-sparing surgery, while 15 (9.3%) required amputation. A complete response was seen in only 18 (11.1%) patients. The necrosis rate was 90% or more in 61 (37.7%) patients. A total of 92.6% patients had negative surgical margins, and 88.3% of patients received adjuvant treatment, while 11.7% did not. Among these, three patients were found to have metastatic disease during adjuvant therapy planning and were transitioned to first-line systemic treatment. Additionally, three patients were unable to receive adjuvant treatment due to intercurrent infections and prolonged hospitalizations, while thirteen patients declined adjuvant therapy despite medical recommendations. There were 100 (61.7%) patients with recurrence during follow-up. While 65% of the cases with recurrence presented as distant metastases, 17% were local recurrences. Additionally, 18% of patients experienced both local recurrence and distant metastases. The most common distant metastasis localization was lung (Table 1).

### 3.2. Survival Analysis

**Figure 1:**
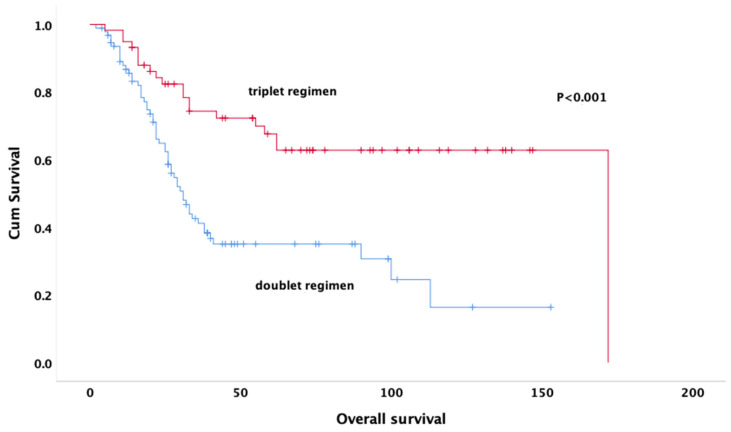
Kaplan–Meier curves for overall survival by treatment groups.

The follow-up period had a mean duration of 86 months (95% CI: 73.8–99.1 months). The median DFS was 9.7 months (95% CI: 7.5–11.8 months). A subgroup analysis was performed to compare survival outcomes between patients receiving MAP (16.7%) and PEI (19.8%) regimens. The results showed no significant difference in OS between the two groups (*p* = 0.794), supporting the inclusion of both regimens under the ’triplet-agent therapy’ category. The median overall survival was 31.0 (25.5–36.4) months for those treated with the doublet regimen and 172 (not reached) months for those treated with the triplet regimen (Figure 1).

At the end of the fifth year, compared to 47.4% of those treated with triple regimen, 13.8% of those treated with doublet regimen were alive (*p* < 0.001).

### 3.3. Univariable and Multivariable Cox Proportional Hazards Models

The univariate analysis showed that age (*p* = 0.011), surgical margin status (*p* = 0.040), and whether patients received adjuvant therapy (*p* < 0.001) were significantly associated with DFS. In multivariate analysis, age (HR: 0.50; 95% CI 0.27–0.92) and receipt of adjuvant treatment (HR: 2.37; 95% CI 1.38–4.06) were significant predictors of DFS (Table 2).

**Figure 2:**
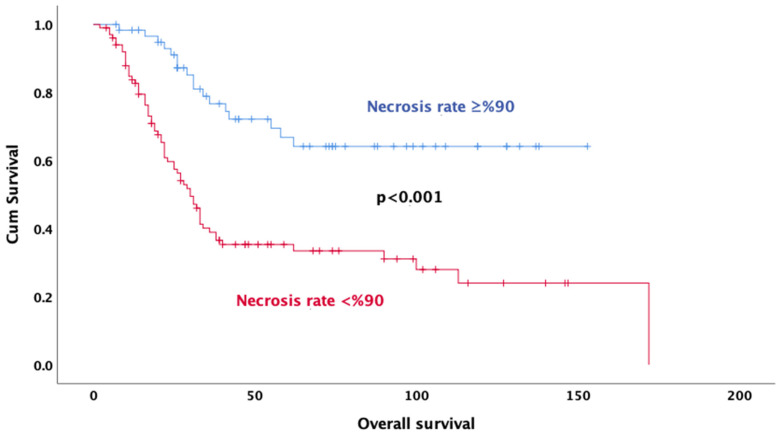
Kaplan–Meier survival curves for overall survival based on necrosis rate.

**Figure 3:**
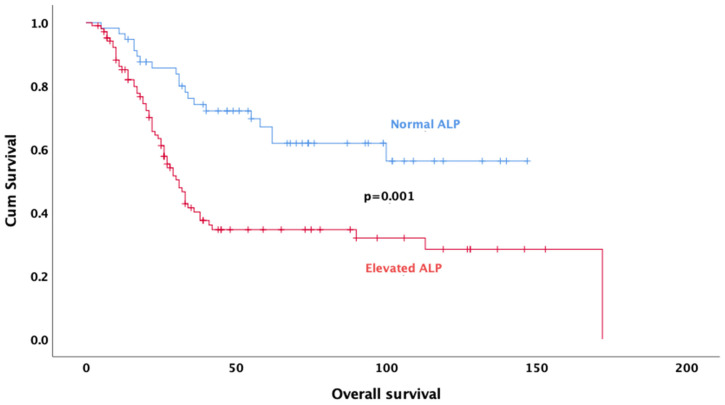
Kaplan–Meier survival curves for overall survival based on ALP levels.

Univariate analysis revealed that age (*p* < 0.001), gender (*p* = 0.032), tumor size (*p* = 0.048), neoadjuvant treatment regimen (*p* < 0.001), pathologic response (*p* = 0.011), rate of necrosis (*p* < 0.001) (Figure 2), alkaline phosphatase (*p* = 0.001) (Figure 3), surgical margin status (*p* = 0.012), and pelvic location (*p* = 0.013) were significantly associated with OS. In multivariate analysis, the results indicate that doublet or triplet neoadjuvant treatment regimen (HR: 1.87; 95% CI 1.07–3.20), necrosis rate above 90% (HR: 0.36; 95% CI 0.20–0.64), ALP elevation (HR: 0.35; 95% CI 0.20–0.64), surgical margin status (HR: 0.34; 95% CI: 0.14–0.82), absence of metastasis at diagnosis (HR: 0.37; 95% CI 0.21–0.67), and gender (HR: 0.59; 95% CI 0.35–0.98) remained significant predictors of OS (Table 3).

## 4. Discussion

We analyzed the demographic, clinical, and pathologic factors affecting DFS and OS in patients with osteosarcoma who received neoadjuvant therapy followed by surgery. The findings of the study elucidate several prognostic factors that affect the treatment response and OS of this patients groups. Receiving adjuvant chemotherapy and being under 18 years of age significantly prolonged the DFS of patients in both univariate and multivariate analysis. These findings emphasize the importance of adjuvant treatment strategies in the treatment process of patients. Our results show that negative surgical margins, necrosis rate of 90% or higher, use of triplet regimen in neoadjuvant treatment, absence of metastatic disease at diagnosis, female gender, and alkaline phosphatase levels in the normal range are significantly associated with OS. These findings are important criteria for optimizing treatment approaches and prognostic evaluation of patients. In particular, the survival-enhancing effects of high necrosis rates and negative surgical margins play a critical role in determining targets for improving treatment processes.

The survival of patients with osteosarcoma has been dramatically improved by intensive systemic chemotherapy and surgery for operable disease [11,12,13]. There are many clinical and methodological studies on neoadjuvant chemotherapy in the literature [14,15]. Multiagent chemotherapy has been used in patients with osteosarcoma since the 1970s, but the most effective chemotherapy regimen has not yet been defined [16]. Currently, the standard approach involves partial chemotherapy before the surgery to facilitate organ-preserving surgery, assess the efficacy of chemotherapeutic agents, prevent pathologic fracture, and eliminate micro metastases [17,18]. In a meta-analysis of nine studies evaluating treatment in osteosarcoma patients, five-year OS rates were shorter in two-drug regimens compared to those containing three or more drugs, at 62% and 70%, respectively. According to the meta-analysis, three-drug regimens containing MAP had significantly better outcomes [19]. Similarly to these results, in our study, the survival of patients receiving triple therapy regimens was longer than that of patients receiving doublet regimens. However, in our study, in addition to the MAP regimen, the PEI regimen, which is commonly used in pediatric and young adult age groups, was also included as a triple regimen. Due to the limited number of patients receiving each regimen separately and the fact that PEI was predominantly administered in the pediatric population, we analyzed triple regimens together rather than separately. Although this approach may limit the ability to compare MAP and PEI regimens directly, it ensures a more robust statistical analysis. The fact that patients receiving the doublet regimen had significantly worse survival rates than those receiving the triplet regimen demonstrates the importance of the effectiveness of the treatment protocols. Our results support the importance of using an intensified chemotherapy regimen in patients with good treatment tolerance, in line with the literature.

In a meta-analysis of 18,126 osteosarcoma patients, male gender, advanced age, large tumor size, extra-extremity osteosarcoma, proximal osteosarcoma, poor response to chemotherapy, and amputation surgery were associated with poor prognosis [20]. Tumor histopathological responses to neoadjuvant chemotherapy are among the strongest prognostic factors in patients with osteosarcoma [21]. Traditionally, response to chemotherapy was described in terms of tumor necrosis [22]. A study of adult osteosarcoma patients evaluating the efficacy of high-dose chemotherapy confirmed that histological response is an important prognostic factor, as patients with 90% or more tumor necrosis had significantly better overall survival than patients with less tumor necrosis [23]. In a study of patients with extremity osteosarcoma, patients with 90% or more necrosis in their surgical specimens had a five-year survival rate close to 80%, which was significantly longer than patients with a necrosis rate less than 90% [12]. Another study by Harting showed a significantly lower OS level found in patients with a necrosis rate of less than 50% [24]. A cohort of 1580 patients supported the use of a necrosis threshold of more than 90% and showed a strong association with survival [25]. O’Kane et al. (2015) reported that patients with tumor necrosis above 90% had a 5-year survival rate of 82%, while those with necrosis rates below 90% had a survival rate of 68% [26]. In our analysis, consistent with the literature, a necrosis rate above 90% was significantly associated with longer survival in osteosarcoma patients receiving neoadjuvant chemotherapy. A high rate of necrosis reflects the sensitivity of tumor cells to treatment and is associated with a more favorable clinical course in patients who respond well to chemotherapy.

Distant metastases remain the main factor affecting patient survival, even after current standard surgical interventions have been applied [27]. A recent study analyzed risk factors and prognostic factors and developed nomograms to assess the risk of distant metastasis in osteosarcoma patients and prognosis in patients with distant metastasis [28]. In a study conducted in Turkey, the five-year OS rate was 20% in the group that had metastases at the time of diagnosis and could be operated after neoadjuvant treatment, while it was 68.3% in the group without metastases and there was a significant difference between the two groups [29]. In another study of 1500 patients, the factors with the largest HR for mortality were metastatic disease at presentation and skip metastasis, with a more than twofold increased risk of mortality [25]. In another study evaluating prognostic factors and histopathologic response analysis to neoadjuvant chemotherapy in osteosarcoma, metastasis at diagnosis was seen as one of the independent prognostic factors and was associated with worse survival [29]. Similarly to other studies, patients with metastatic disease at diagnosis had shorter overall survival in our study. As these results suggest, although these patients who received neoadjuvant chemotherapy became eligible for surgical intervention, the presence of metastatic disease is a critical factor that negatively affects the response to treatment. Although neoadjuvant treatment improves surgical success by reducing the size of tumors, systemic disease burden is an important factor affecting post-treatment survival rates. One of the reasons for this is probably that patients with metastases tend to be resistant to intensive treatment. These results support the aggressive nature of the disease, and the presence of metastasis complicates the treatment process.

Most studies have reported a difference in prognosis between patients with positive and negative surgical margins. There are studies showing that patients with high-grade osteosarcoma of the extremity with positive surgical margins have a higher probability of local recurrence compared with those with negative surgical margins. These studies also found the results to be associated with poorer survival [30,31]. There are also studies in the literature showing that close surgical margins do not increase the risk of local recurrence in osteosarcoma [32]. This result is probably attributed to the effect of preoperative chemotherapy to kill microsatellite lesions beyond the primary tumor [32]. In our study, although all patients received neoadjuvant treatment, survival time in patients with positive surgical margins was shorter than in patients with negative surgical margins. Based on this information, we can say that negative surgical margins are associated with better post-treatment response and reduced recurrence rates. Therefore, the importance of rigorous evaluation of surgical margins, multidisciplinary approaches and preoperative planning to increase the effectiveness of surgical intervention in osteosarcoma is emphasized once again.

ALP, as a biomarker reflecting activity in bone metabolism, can provide information about tumor burden and disease aggressiveness in osteosarcoma. The significance of serum ALP level as a prognostic factor of chemotherapy response might also be considered. This can be associated with the presence of osteoblast transformation and osteoclast activation, which will further interfere with the control of cell differentiation, proliferation, and degradation. These conditions result in elevated serum ALP levels [33,34]. A study evaluating osteosarcoma patients receiving neoadjuvant therapy showed a significant correlation between high post-treatment serum ALP levels and low survival rate [35]. In another study by Han et al., high ALP levels were associated with worse survival in osteosarcoma patients [36]. ALP levels in the normal range indicate that the disease is better controlled, and the prognosis may be favorable, and consistent with these results, in our study, survival was better in patients with normal ALP levels before systemic therapy. These findings emphasize the importance of ALP level as a prognostic biomarker in osteosarcoma treatment and reveal that it is a factor that should be considered in treatment planning. Therefore, monitoring ALP levels in the clinical follow-up of patients may be critical for prognosis prediction and optimization of treatment strategies.

There are conflicting results suggesting that female gender has a survival advantage in osteosarcoma patients. The Scandinavian Sarcoma Group found sex to correlate with outcome, female patients having fewer relapses and better survival than their male counterparts [37]. A study of 1702 patients found that female gender was associated with a higher probability of a good response on univariate and even multivariate analysis. Unexpectedly, however, this did not translate into a survival advantage for women, despite the strong overall correlation between response and survival [38]. In our study, being female was found to be significantly associated with longer survival. Females’ better survival rates may be due to a variety of factors, including hormonal factors, immune system responses and potential differences in tumor biology. These findings provide an important basis for further investigation of the gender-related biological dynamics of osteosarcoma and optimization of treatment processes.

Our study has some limitations. The number of patients is relatively small. The size and heterogeneity of the patient cohort limits the statistical power of the cohort and may have led to a selection bias that potentially affected the results of the study. Our study included patients whose treatment was initiated in pediatric oncology and followed up in the adult oncology outpatient clinic. Since we did not have access to the information of the entire cohort in the pediatric age group, we do not have good information about the survival of the pediatric group. There may be a bias because the patients included in our follow-up were patients who responded well to treatment and were admitted to our outpatient clinic. Consequently, our survival outcomes may be overestimated, as patients who did not survive into adulthood were not captured in our analysis. This limitation should be taken into account when interpreting the results, especially in comparison with studies including the full pediatric cohort. Another limitation is the inclusion of both MAP and PEI regimens in the triple therapy group, as the limited number of patients in each subgroup prevented separate statistical analysis. Moreover, the PEI regimen was predominantly used in the pediatric population, complicating direct comparisons between regimens. Since the patients in our study were from different institutions, they were not operated on by a single surgeon and treatment modalities were not standardized in the management of the disease. Despite our efforts to reach out to patients and review medical records, missing data such as chemotherapy doses and radiotherapy doses and duration for patients receiving radiotherapy posed a significant challenge. Additionally, although our study evaluated ALP levels, tumor size, and metastatic status as prognostic determinants, we acknowledge that other biological markers, such as LDH levels and genetic mutations, may also influence survival outcomes. However, due to the retrospective nature of this study, comprehensive data on these parameters were not systematically available. Given the emerging evidence on the prognostic significance of molecular and biochemical markers in osteosarcoma, future studies integrating genomic and metabolic profiling will be essential to refine risk stratification and optimize treatment strategies. Despite these limitations, the present study provides valuable information on prognostic factors affecting survival in patients with osteosarcoma who receive neoadjuvant therapy and undergo surgery.

In conclusion, <90% tumor necrosis, use of doublet regimen in neoadjuvant treatment, presence of metastatic disease at diagnosis, high ALP level at the beginning of the treatment regimen, positive surgical margin and male gender significantly deteriorated the survival of the patients and were found to be independent prognostic factors. These findings highlight the critical importance of recognizing and addressing these prognostic factors in the management of osteosarcoma, paving the way for more personalized therapeutic approaches that aim to enhance patient outcomes and improve survival rates.

## Figures and Tables

**Table 1:** Demographic and clinical characteristics of the study patients.

Age, Year	
Median (IQR)	20 (18–29)
Gender, n (%) Female Male	76 (46.9) 86 (53.1)
ECOG-PS, n (%) 0–1 ≥2	160 (98.8) 2 (1.2)
Histology, n (%) Osteoblastic Chondroblastic Fibroblastic Telangiectatic NOS	66(40.7) 46(28.4) 16 (9.9 12 (7.4) 22 (13.6)
Tumor size <8 cm ≥8 cm	49 (30.2) 113 (69.8)
Location, n (%) Lower extremity Upper extremity Axial/vertebra	135 (83.3) 18 (11.1) 9 (5.6)
Neoadjuvant treatment, n (%) Cisplatin + doxorubicin (doublet regimen) PEI (Triplet regimen) MAP (Triplet regimen) Others	94 (58) 32 (19.8) 27 (16.7) 9 (5.5)
Type of surgery, n (%) Limb-sparing surgery Amputation	147 (90.7) 15 (9.3)
Pathologic response, n (%) Complete response Presence of residual	18 (11.1) 144 (88.9)
Rate of necrosis ≥%90, n (%) Doublet Treatment Triplet Treatment	25 (43.85) 32 (56.15)
Surgical margin, n (%) R0 R1/2	150 (92.6) 12 (7.4)
Adjuvant therapy, n (%) Yes No	143 (88.3) 19 (11.7)
LDH, n (%) Elevated levels Normal levels	72 (44.4) 90 (55.6)
ALP, n (%) Elevated levels Normal levels	105 (64.8) 57 (35.2)
Metastases at diagnosis, n (%) No metastasis Skip metastasis (Bone only) Distant metastasis (lung)	136 (84) 8 (4.9) 18 (11.1)
Progression in follow-up, n (%) Local recurrence Distant metastasis Local recurrence + distant metastasis	17 (17.0) 65 (65.0) 18 (18.0)

IQR: Interquartile range; ECOG-PS: Eastern cooperative oncology group-performance status; LDH: Lactate dehydrogenase; ALP: Alkaline phosphatase. Skip metastases were defined as non-contiguous tumors spread within the same bone or adjacent bone without distant organ involvement. These cases did not preclude surgery.

**Table 2:** Clinical and pathological factors related to disease free survival.

	Univariate		Multivariate	
	Median DFS	*p*	HR (%95 CI)	*p*
Age <65 ≥65	4.0 (2.1–5.9) 4.0 (2.8–5.1)	0.697	Ref 0.50 (0.27–0.92)	0.024
Gender Female Male	3.0 (2.3–3.7) 4.0 (2.8–5.1)	0.955		
Tumor size <8 cm ≥8 cm	9.4 (2.1–16.7) 9.7 (7.9–11.5)	0.717		
Neoadjuvant treatment Triplet regimen Doublet regimen	12.7 (9.9–15.6) 8.6 (6.4–10.8)	0.091		
Pathologic response Complete response Presence of residual	4.1 (1.4–6.7) 3.4 (2.5–4.2)	0.340		
Rate of necrosis %90 and above <90	13.7 (11.2–16.1) 8.6 (6.8–10.5)	0.063		
Alkaline phosphatase Normal levels Elevated levels	12.8 (10.5–15.2) 8.6 (6.3–10.9)	0.301		
Lactate dehydrogenase Normal levels Elevated levels	12.4 (8.8–16.0) 8.6 (6.1–11.1)	0.292		
Surgical margin R1/2 R0	2.7 (0.1–5.3) 10.4 (7.5–13.2)	0.040	Ref 0.62 (0.32–1.21)	0.163
Adjuvant therapy Yes No	12.3 (9.5–15.0) 3.4 (1.0–5.6)	<0.001	Ref 2.37 (1.38–4.06)	<0.001
Pathological fracture No Yes	10.0 (6.9–12.9) 8.6 (4.6–12.7)	0.320		
Pelvic location Yes No	5.5 (1.9–9.1) 10.6 (7.2–14.0)	0.094		
Metastases at diagnosis Yes No	7.1 (2.5–11.6) 10.4 (7.3–13.4)	0.363		

DFS: Disease-free survival; HR: Hazard ratio; CI: Confidence interval.

**Table 3:** Clinical and pathological factors related to overall survival.

	Univariate		Multivariate	
	Median OS	*p*	HR (%95 CI)	*p*
Age <18 ≥18	172.0 (NR) 33.0 (27.1–38.9)	<0.001	Ref 0.53 (0.22–1.3)	0.163
Gender Male Female	33.0 (23.0–42.9) 113.0 (NR)	0.032	Ref 0.59(0.35–0.98)	0.040
Tumor size <8 cm ≥8 cm	NR 38.0 (18.7–57.2)	0.048	Ref 1.20 (0.71–2.03)	0.490
Neoadjuvant treatment Triplet regimen Doublet regimen	172.0 (NR) 31.0 (25.6–36.4)	<0.001	Ref 1.87 (1.07–3.20)	0.021
Pathologic response Complete response Presence of residual	NR 36.0 (18.0–54.0)	0.011	Ref 0.86 (0.26–2.78)	0.812
Rate of necrosis <90% ≥90%	30.0 (25.2–34.8) NR	<0.001	Ref 0.36 (0.20–0.64)	<0.001
Alkaline phosphatase Elevated levels Normal levels	31.0 (26.0–35.9) NR	0.001	Ref 0.35 (0.20–0.64)	<0.001
Lactate dehydrogenase Normal levels Elevated levels	62.0 (10.4–113.5) 33.0 (23.2–42.7)	0.091		
Surgical margin R1/R2 R0	17.0 (8.5–25.4) 55.0 (14.3–95.7)	0.012	Ref 0.34 (0.14–0.82)	0.010
Pathological fracture No Yes	58 (10.2–105.8) 33 (25.8–40.2)	0.272		
Pelvic location No Yes	55.0 (3.9–106.0) 22.0 (11.7–32.2)	0.013	Ref 0.79 (0.38–1.62)	0.523
Metastases at diagnosis Yes No	31.0 (17.2–44.8) 62.0 (6.0–117.9)	<0.001	Ref 0.37 (0.21–0.67)	0.010

OS: Overall survival; HR: Hazard ratio; CI: Confidence interval; NR: Not reached.

## Data Availability

The data presented in this study are available on request from the corresponding author.
